# The effect of old-age pensions on health care utilization patterns and insurance uptake in Mexico

**DOI:** 10.1136/bmjgh-2019-001771

**Published:** 2019-11-12

**Authors:** Carlos Riumallo-Herl, Emma Aguila

**Affiliations:** 1 Applied Economics, Erasmus Universiteit Rotterdam, Rotterdam, Netherlands; 2 Sol Price School of Public Policy, University of Southern California, Los Angeles, California, USA

**Keywords:** health economics, health insurance, health policy, other study design

## Abstract

**Introduction:**

As old-age pensions continue to expand around the world in response to population ageing, policymakers increasingly wish to understand their impact on healthcare demand. In this paper, we examine the effects of supplemental income to older adults on healthcare use patterns, expenditures and insurance uptake in Yucatan, Mexico.

**Method:**

We use a longitudinal survey for individuals aged 70 or older and an individual fixed-effects difference-in-difference approach to understand the effect of an income supplement on healthcare use patterns, out-of-pocket expenditures and health insurance uptake patterns.

**Results:**

The implementation of the old-age pension was associated with increased use of healthcare with nuanced effects on the type of care. Old-age pensions increase the use of formal healthcare by 15 percentage points (95% CI 6.1 to 23.9) for those with healthcare use at baseline and by 7.5 percentage points (95% CI 3.7 to 11.3) for those without healthcare use at baseline. We find no evidence of greater out-of-pocket expenditures, likely because old-age pensions were associated with a 4.2 percentage point (95% CI 1.5 to 6.9) increase in use of public health insurance.

**Conclusion:**

Old-age pensions can shift healthcare demand towards formal services and eliminate financial barriers to basic care. Pension benefits can also increase the uptake of insurance programmes. These results demonstrate how social programmes can complement each other This highlights the potential role of old-age pensions in achieving universal health coverage for individuals at older ages.

**Key questions**

**What is already known?**

- Old-age pensions lead to increased use of overall healthcare but also higher out-of-pocket expenditures in low and middle-income countries.
- There is little information on the interactions between social programmes in improving universal healthcare.
- Old-age pensions are frequently used by policymakers to reduce poverty at older ages.

**What are the new findings?**

- Old-age pensions have a heterogeneous effect on types of healthcare used. Old-age pensions encourage the use of formal healthcare for those with previous use and use of informal healthcare for those without previous use.
- Higher use does not necessarily lead to higher out-of-pocket expenditures because old-age pensions also lead to a higher uptake of health insurance.
- The interactions between social programmes, such as old-age pensions and public health insurance, can enhance universal access to healthcare while providing financial protection.

**What do the new findings imply?**

- Old-age pension policies can reduce poverty and encourage the uptake of other social programmes such as public health insurance.
- Old-age pension programme design should consider complementarity with other programmes.
- Old-age pensions should be considered as another mechanism to achieve universal healthcare coverage.

## Introduction

In recent decades, many low and middle-income countries have implemented social protection programmes to provide income support for the elderly.^1–3^ The financial costs of these programmes require a deeper understanding of how old-age pensions and conditional cash transfers impact individual well-being. Several studies have evaluated the effect of old-age pensions on myriad outcomes.^4–6^ Previous studies have analysed the effect of different programmes, with old-age pension programmes being identified as an important means for improving individual well-being.

Health is among the outcomes that social programmes may improve. Gertler^5^ found that conditional cash transfers led to improvements in child health. Other studies have also found benefits on elderly health.^7 8^ Little, however, is known about the mechanisms by which old-age pensions improve individual health. One possibility is through nutrition. Evidence from the last three decades has shown how higher caloric intake is often associated with better population health.^9–11^ Such an effect may only be evident in the long term,^12^ leaving unanswered the question of whether cash transfers improve health in the short term.

A mechanism that may improve healthcare in the short term is healthcare utilisation. Previous research has shown a weak link between healthcare utilisation and better health. For example, the RAND health insurance experiment found only small health effects from greater healthcare use.^13^ This may be because a marginal increase in income in developed countries can only lead to an increased use of care that is low in cost-effectiveness.^14^ In low and middle-income countries, a marginal increase in income could lead to access to more cost-effective care. Previous research does not indicate how greater income can affect different dimensions of healthcare in low and middle-income countries, with most studies only looking at overall healthcare use.

In this paper, we use an ageing survey and a difference-in-difference approach to exploit the experimental implementation of an old-age pension programme in Mexico. We study the impact of old-age cash transfers on three dimensions of healthcare: utilisation, expenditures and insurance uptake. We further disaggregate the results by healthcare use and insurance coverage at baseline to understand how pensions impact different segments of the elderly population. We also examine complementarities between the old-age pension and public health insurance. Our findings suggest that cash transfers can shift healthcare utilisation from low to high-quality services, eliminate financial barriers for basic care and encourage enrolment in public health insurance schemes. Our study provides further insights on how cash transfers may improve health in low and middle-income countries^15 16^ and highlights the interactions between different social programmes.

There are few studies that evaluate the effect of unremarkable pensions on healthcare utilisation patterns, out-of-pocket (OOP) health expenditures and uptake of health insurance in low and middle-income countries. Two previous studies in Brazil found that pensions improve household access to healthcare services and medications but may not change individual use by the elderly.^17 18^ A previous study in Mexico finds pensions are associated with a higher proportion of elderly recipients reporting visits to doctors^19^ but does not evaluate effects on different types of care.

Our results show that an unremarkable pension encourages the use of formal healthcare and the likelihood of taking prescribed medications among the elderly. We also find that older individuals with no use of healthcare at baseline begin to use informal care services. This suggests that old-age pensions can eliminate financial barriers to basic care. Individuals with prior use of healthcare shift from informal to formal services, suggesting a move from low-quality to high-quality healthcare once minimum needs are covered. A shift from low to high-quality care may help explain improvements in health found in previous studies of cash transfers.^15 16 20^ At the same time, we find no evidence of increased health expenditures, an outcome explained mainly by the increased uptake of health insurance. Together, these results demonstrate interactions and complementarities that can exist between government-sponsored programmes.

## Methods

In this study we exploit the experimental implementation of the *Reconocer* pension programme in Mexico. This scheme was implemented in Yucatan to address the growing poverty rate among older adults in the state. The programme was implemented in three phases over 37 localities in the state of Yucatan, Mexico, during a 4-year period.^21^ Phase I and phase II were implemented in rural areas, where the programme provided a monthly food basket and cash payments of MXN$500 (US$55.92 in 2019 purchasing power parity, or PPP) to adults aged 70 and older. The programme was expanded to urban areas in phase III, where its main component was a monthly cash transfer of MXN$550 (US$61.52 in 2019 PPP), an amount approximately one-half of the monthly minimum wage in Yucatan, MXN$1089 per month or US$121.80 in 2019 PPP.^22^

During the third phase, *Reconocer* expanded to cities with populations of more than 20 000 inhabitants. These cities were Hunucma, Kanasin, Uman, Merida, Motul, Oxcutzcab, Progreso, Tekax, Ticul, Tizimin and Valladolid. A cluster randomised controlled trial design was conducted among these cities, with a pairwise matching procedure ensuring similarity across treatment and control locations in household and other community indicators from the 2005 Census. One of the matched pairs was chosen randomly and, within that pair, one of the localities, Valladolid, was randomly allocated as a treatment group and the other, Motul, as a control. The first pension payment was made in Valladolid in December 2008.^21^ Payments were distributed at the town hall or delivered to those who were unable to go to the town hall because of their health. Valladolid and Motul were not only similar to each other, they were similar to the median village of 20 000–50 000 inhabitants in Mexico. They are not extreme outliers but instead represent many similar locations in Mexico, as well as in other low and middle-income countries (online supplementary appendix table 1).

10.1136/bmjgh-2019-001771.supp1 Supplementary data

For this study we use data from the baseline and follow-up waves of the Survey of Household Economic Characteristics in the State of Yucatan (ENCAHEY) conducted by the RAND. As such, our analysis constitutes secondary use of data and did not involve recruitment or involvement of patients. The survey collected data on a random sample of individuals aged 70 and older by face-to-face interviews 3 months prior to and 6 months after programme implementation. Online supplementary appendix table 2 shows that the number of individuals included in this study was similar to that enumerated in the census, demonstrating that the survey was representative of individuals aged 70 and older in these locations.

The survey is comparable to the Mexican Health and Aging Study (MHAS) but was adapted to reflect the cultural and institutional characteristics of Yucatan. It included a comprehensive set of measures covering health, demographics, household income and household consumption. Validated measures for each of these domains were adapted from existing surveys such as the MHAS; the US Health and Retirement Survey; the US Panel Study of Income Dynamics; and the Survey on Health, Well-Being, and Aging in Latin America and the Caribbean, among others. Complete descriptions of the protocols are available elsewhere.^23^

Our final sample consisted of 2324 individuals aged 70 or older. The response rates at baseline were 91.4% in Valladolid and 95.2% in Motul; the follow-up response rates were 87.9% in Valladolid and 81.9% in Motul. Table 1 presents sample characteristics at baseline for each location. It shows that both villages are comparable, except for gender. Valladolid, the treatment location, has a lower fraction of males in the sample than Motul, the control location. Average age is approximately 78 years old and the average level of schooling is 1.8 years. Most individuals are either in a partnership (52%) or widowed (39%). Average household size is 3.4 persons and average monthly income per capita is approximately US$100. A similar fraction in both cities worked for pay in the 3 months prior to the baseline survey. A higher fraction of individuals in Motul reported chronic conditions, but there were no significant differences between the two locations in the number of daily activity limitations. Table 1 also shows that 86% of individuals in Valladolid reported receiving the *Reconocer* pension in the second wave. While the locations differed in follow-up response rate, online supplementary appendix tables 3 and 4 show no systematic attrition differences in either location.

**Table 1:** Summary statistics of demographic and health status variables

	Control (1)	Treatment (2)	Difference (1)–(2)	P value
Male	0.515	0.466	−0.048	0.022
	0.500	0.499		
Age	77.800	77.857	0.057	0.835
	6.405	6.582		
Education (years)	1.906	1.792	−0.114	0.142
	1.727	1.931		
Marital status: couple	0.523	0.524	0.001	0.949
	0.500	0.500	.	
Marital status: divorced or separated	0.027	0.034	0.008	0.287
	0.161	0.182		
Marital status: widow	0.391	0.386	−0.005	0.827
	0.488	0.487		
Household size	3.446	3.429	−0.017	0.848
	2.082	2.160		
Works for pay	0.153	0.166	0.013	0.414
	0.360	0.372		
Household monthly income per capita (December 2008 US$)	98.508	105.707	7.199	0.141
	78.795	137.357		
Any chronic conditions	0.645	0.582	−0.063	0.077
	0.848	0.826		
IADL	4.819	4.666	−0.153	0.358
	3.788	3.840		
Receives *Reconocer* pension in wave 2	0.000	0.860	0.860	0.000
	0.000	0.347		
Observations	973	1307		

Values represent means or fractions. SDs are presented below means.

IADL, Instrumental Activities of Daily Living.

### Healthcare demand: utilisation, expenditures and insurance uptake

We evaluated three dimensions of healthcare: utilisation, expenditures and insurance uptake. For healthcare utilisation, we combine different types of self-reported care into the following categories: formal, informal, hospital, medication adherence and access to any type of care. We define formal healthcare as using dental care, outpatient care or visiting a doctor in the 3 months before the survey. Informal care corresponds to the use of folk healers and pharmacists in the same time period. Hospital care utilisation is defined as having at least one hospital stay during the 3 months prior to the survey. We define medication adherence as taking medication conditional on having a prescription. For healthcare expenditures, we self-reported expenditures for the 3 months prior to the survey for formal care, informal care and hospital care, excluding medications for which there are no expenditure data. We use the natural logarithm of expenditures to account for non-linearities and outliers. Finally, for health insurance, we define four categories based on the characteristics of the Mexican healthcare system: private health insurance, social security, public health insurance and any health insurance. Private health insurance, social security and public health insurance are mutually exclusive categories. Any health insurance is defined as having any one of the three schemes of health insurance. The proportion of persons having any health insurance is the inverse of the proportion not having health insurance.

### Statistical analysis

To estimate the effect of old-age pensions, we use an individual fixed-effects difference-in-difference approach. These models exploit the differential implementation of the programme across villages while accounting for time-invariant individual characteristics. More specifically, the treatment group corresponds to all individuals at least 70 years of age and residing in Valladolid, where the pension programme was implemented, and the control group represents all individuals of that age in Motul. An advantage to this approach is the randomisation of treatment across villages.

The identifying assumption of the difference-in-difference approach is the common trends assumption, which is shown to hold in this case. The ENCAHEY unfortunately does not provide preintervention waves to evaluate the common trends assumption, but we use data from the Mexican census and the Encuesta Nacional de Ingresos y Gastos de Hogares survey to do so. Online supplementary appendix figures 1-4 and appendix tables 5-7 show that Valladolid and Motul had similar demographic, health insurance coverage, health expenditure and poverty trends prior to programme implementation. Following the recommendations of previous research, we evaluated the robustness of our results by using a matching difference-in-difference approach.^24^ This approach matched individuals from both cities according to their baseline propensity score based on age, gender, education, marital status, per capita household income, insurance and healthcare use (online supplementary appendix figure 5). The matching led to a successful pairing of 805 individuals from Motul and 1006 individuals from Valladolid and improved the balance obtained before. This matching difference-in-difference approach allowed us to verify the robustness of our results. To appropriately estimate the SEs, we present bootstrapped SEs as recommended in previous research.^25^

Another necessary condition for our analysis method is that no other reforms were implemented differentially during the evaluation period. Two relevant programmes to consider here are *Oportunidades* and *Seguro Popular. Oportunidades*, a cash transfer programme implemented in 1997 (as *Progresa*), achieved maximum coverage in 2005.^26^ *Seguro Popular*, the public health insurance programme, had varying roll-out dates across municipalities, but its roll-out was completed in Valladolid and Motul by 2006.^27^ Both programmes were therefore fully implemented in both locations at the time of the pension programme implementation.

Our main and robustness analyses use the following specification:

(1) $$Y_{it}=\beta_{0}+\beta_{1}T_{i}+\beta_{2}W_{2}+\beta_{3}T_{i}W_{2}+\sum_{j=4}^{n}\beta_{j}X_{j,i,t}+I_{i}+\epsilon_{i,t}$$

where $Y_{it}$ is the outcome discussed above; $T_{i}$ is a binary variable indicating whether individual *i* is in the treatment village that receives the old-age pension; $X_{j,i,t}$ are the controlling variables including marital status, age fixed effects, number of years of education, whether the individual receives any other pension income, number of daily living limitations (activities of daily living) and number of chronic conditions; $W_{2}$ is the follow-up wave fixed effect; and $I_{i}$ are individual fixed effects.

For binary outcomes, we estimate the effect of the old-age pensions using a linear probability model. We also conduct some analyses with non-linear logistics models to verify the robustness of our results to the functional assumptions of ordinary least squares (OLS). To appropriately estimate the SEs given the correlation of outcomes within a village, we present bootstrapped SEs as recommended in the literature.^25^

In the analysis below, we evaluate the heterogeneous effect of old-age pensions by estimating the same models but stratifying by baseline use of care and health insurance. We categorise baseline use of care as no use or any use. We categorise by baseline health insurance coverage as having no health insurance or any health insurance. This strategy provides the estimates for the impact of the overall programme and not on individual take-up of old-age pensions. As such, it portrays the impact that such policies can have while considering the existence of non-compliers. Take-up rate of the old-age pension programme was 92%, as reported in administrative records.

### Patient and public involvement

No patient and public were involved in this study. This paper only uses secondary data of an already implemented intervention.

## Results

The results from the individual fixed-effects difference in difference are presented in table 2. Panel A shows that the old-age pension programme is associated with a 6.8 percentage point increase in use of any type of health service (95% CI −0.9 to 11.4), which was driven by a 9.0 percentage point (95% CI 1.5 to 16.4) increase in use of formal healthcare services. We also find that the pension scheme is associated with a 7.7 percentage point (95% CI 2.0 to 13.4) increase in medication adherence. The pension programme is not associated with changes in hospital care (95% CI −1.9 to 4.4) or informal care (95% CI −3.2 to 5.0).

**Table 2:** Effect of the old-age pension programme on healthcare utilisation, health expenditures and insurance uptake

	(1)	(2)	(3)	(4)	(5)
Panel A: healthcare utilisation					
	Formal care	Informal care	Hospital stays	Medication	Any access
Treatment*Post	0.090 (0.015 to 0.164)	0.009 (−0.032 to 0.050)	0.014 (−0.019 to 0.047)	0.077 (0.020 to 0.134)	0.068 (−0.006 to 0.143)
Post	0.042 (−0.261 to 0.345)	0.016 (−0.188 to 0.220)	−0.078 (−0.220 to 0.065)	−0.146 (−0.392 to 0.100)	0.009 (−0.300 to 0.319)
Observations	3818	3818	3817	3815	3818
Individuals	2397	2397	2396	2396	2397
Panel B: healthcare expenditures (log)					
	Formal care	Informal care	Hospital stays	Total OOP	
Treatment*Post	−0.120 (−0.311 to 0.071)	0.024 (−0.040 to 0.089)	−0.013 (−0.109 to 0.082)	−0.100 (−0.305 to 0.105)	
Wave 2	0.841 (0.179 to 1.503)	0.227 (−0.248 to 0.702)	−0.145 (−0.396 to 0.106)	0.975 (0.181 to 1.768)	
Observations	3818	3818	3818	3818	
Individuals	2397	2397	2397	2397	
Panel C: health insurance (HI) uptake					
	Social security	Private HI	Public HI	Any HI	
Treatment*Post	−0.006 (−0.032 to 0.020)	−0.000 (−0.008 to 0.007)	0.042 (0.015 to 0.069)	0.031 (−0.002 to 0.063)	
Wave 2	−0.021 (−0.089 to 0.046)	−0.008 (−0.028 to 0.011)	−0.011 (−0.077 to 0.055)	−0.048 (−0.136 to 0.041)	
Observations	3751	3751	3751	3751	
Individuals	2367	2367	2367	2367	

Confidence intervals were obtained using bootstrapped SEs. The coefficients are estimated with an individual fixed-effects difference-in-difference models controlling for changes in age fixed effects, education years, marital status, household size, labour force status, chronic conditions and number of limitations in daily living activities. Valladolid is the treatment village where the old-age pension programme was implemented in December 2008. The models use data from the baseline and follow-up survey.

OOP, out of pocket.

The results in panel B show that, contrary to economic theory,^28^ the pension programme did not lead to greater expenditures for medical care. The combination of greater healthcare use without increases in expenditures is likely explained by the 3.1 percentage point (95% CI −0.2 to 6.3) increase in uptake of any health insurance. This increase is mainly driven by the 4.2 percentage point (95% CI 1.5 to 6.9) increase in uptake of public health insurance. We reach similar conclusions when using a logistic model for the binary outcomes (online supplementary appendix table 8).

**Figure 1:**
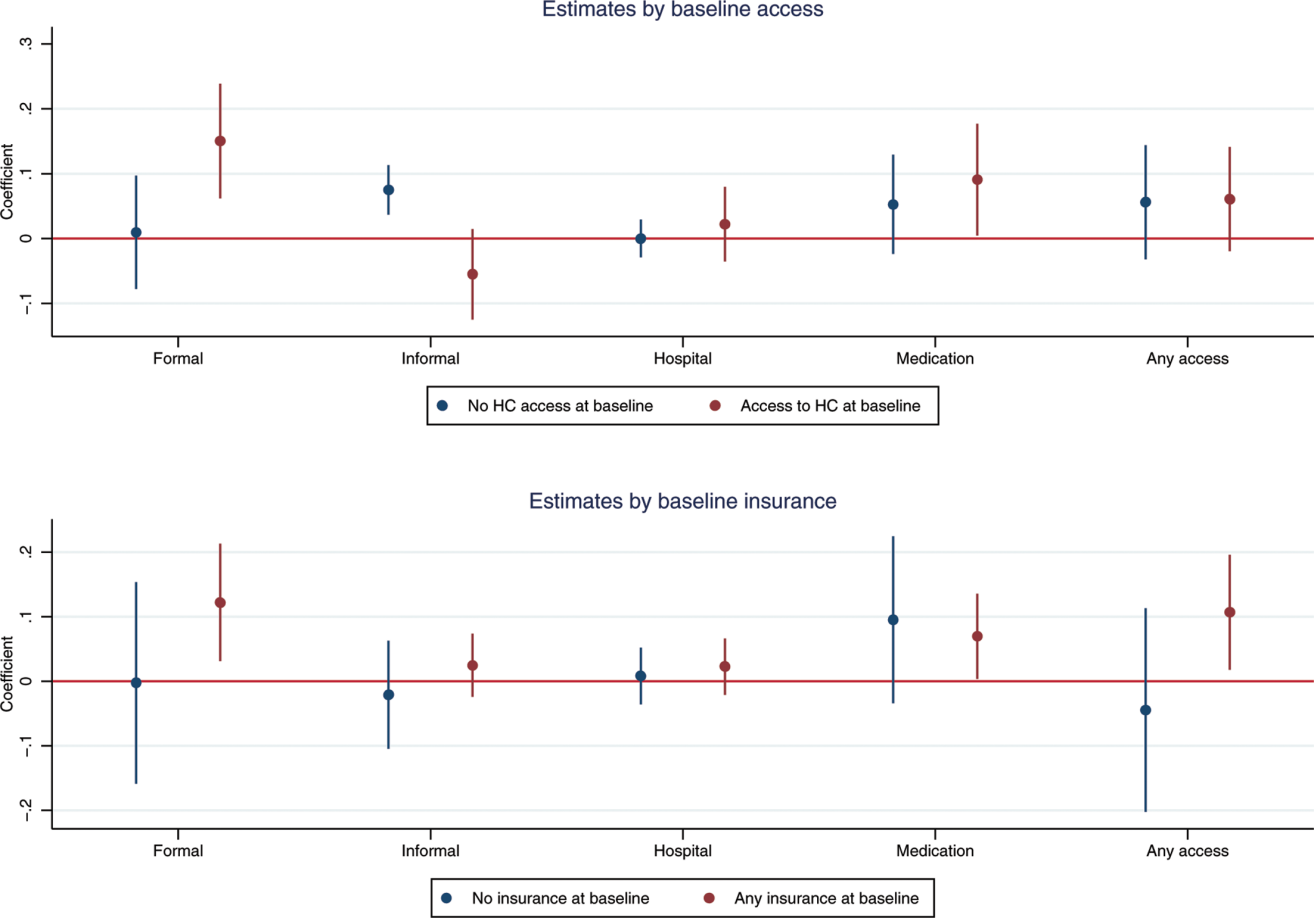
Impact of old-age pension on healthcare utilisation by baseline access and insurance coverage. HC, healthcare.

Our results also suggest important heterogeneities across individuals. Figure 1 presents the estimated coefficients and CIs for the impact of *Reconocer* on healthcare utilisation by baseline use and insurance coverage. The upper panel shows that individuals with any healthcare utilisation at baseline and who subsequently received pensions increased their use of formal services by 15.0 percentage points (95% CI 6.2 to 23.9) and reduced their use of informal services by 5.5 percentage points (95% CI −12.5 to 1.5). Individuals without access at baseline increased their use of informal services by 7.5 percentage points (95% CI 3.7 to 11.3). The lower panel shows the results by baseline insurance status. We find that individuals with health insurance at baseline increased use of any type of healthcare by 10.7 percentage points (95% CI 1.8 to 19.6), formal services by 12.2 percentage points (95% CI 3.1 to 21.3) and medication adherence by 6.9 percentage points (95% CI 0.3 to 13.6). We find no impact of the pension scheme on healthcare for individuals without insurance at baseline.

**Figure 2:**
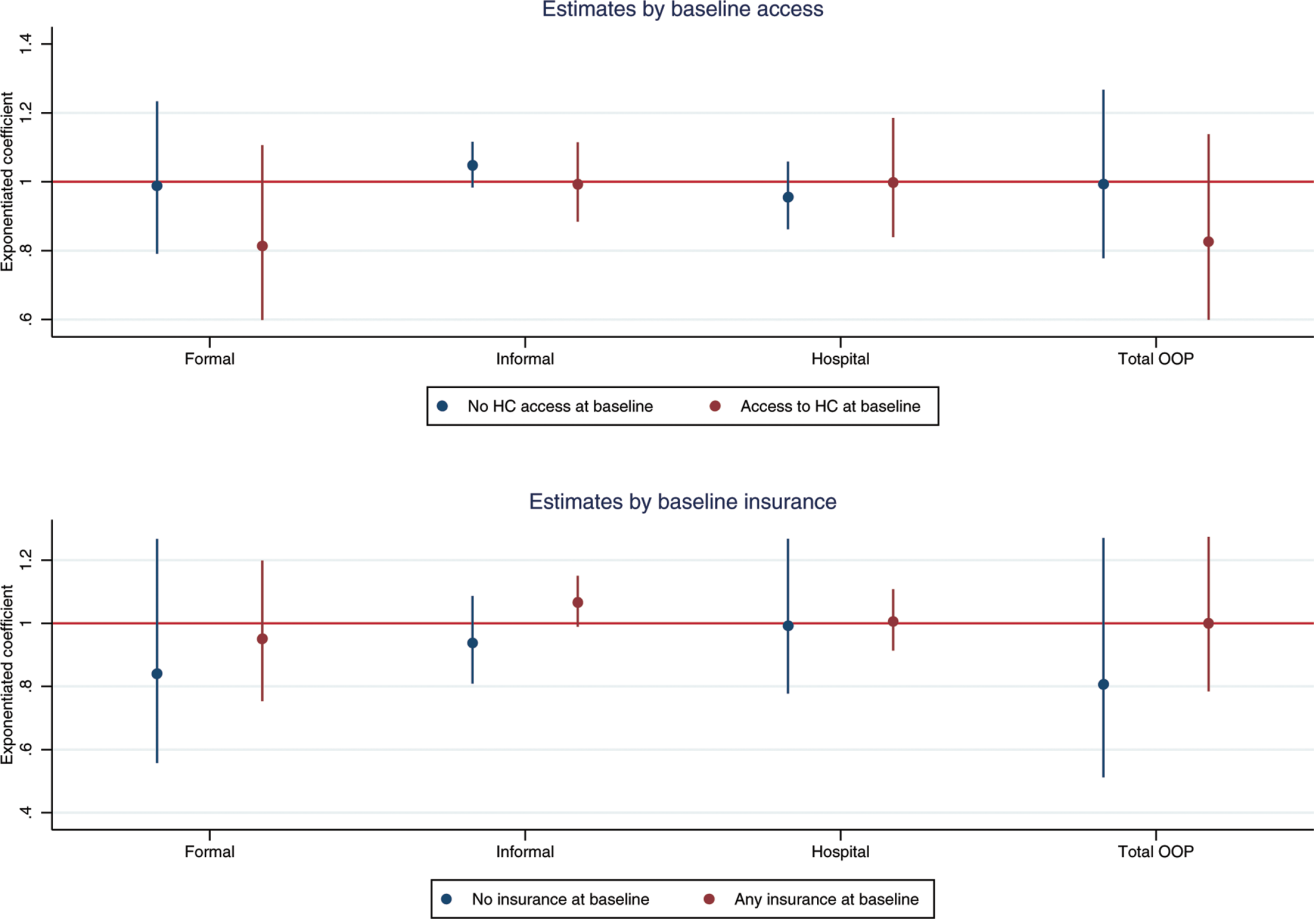
Impact of old-age pensions on healthcare expenditures by baseline access and insurance coverage. HC, healthcare; OOP, out of pocket.

**Figure 3:**
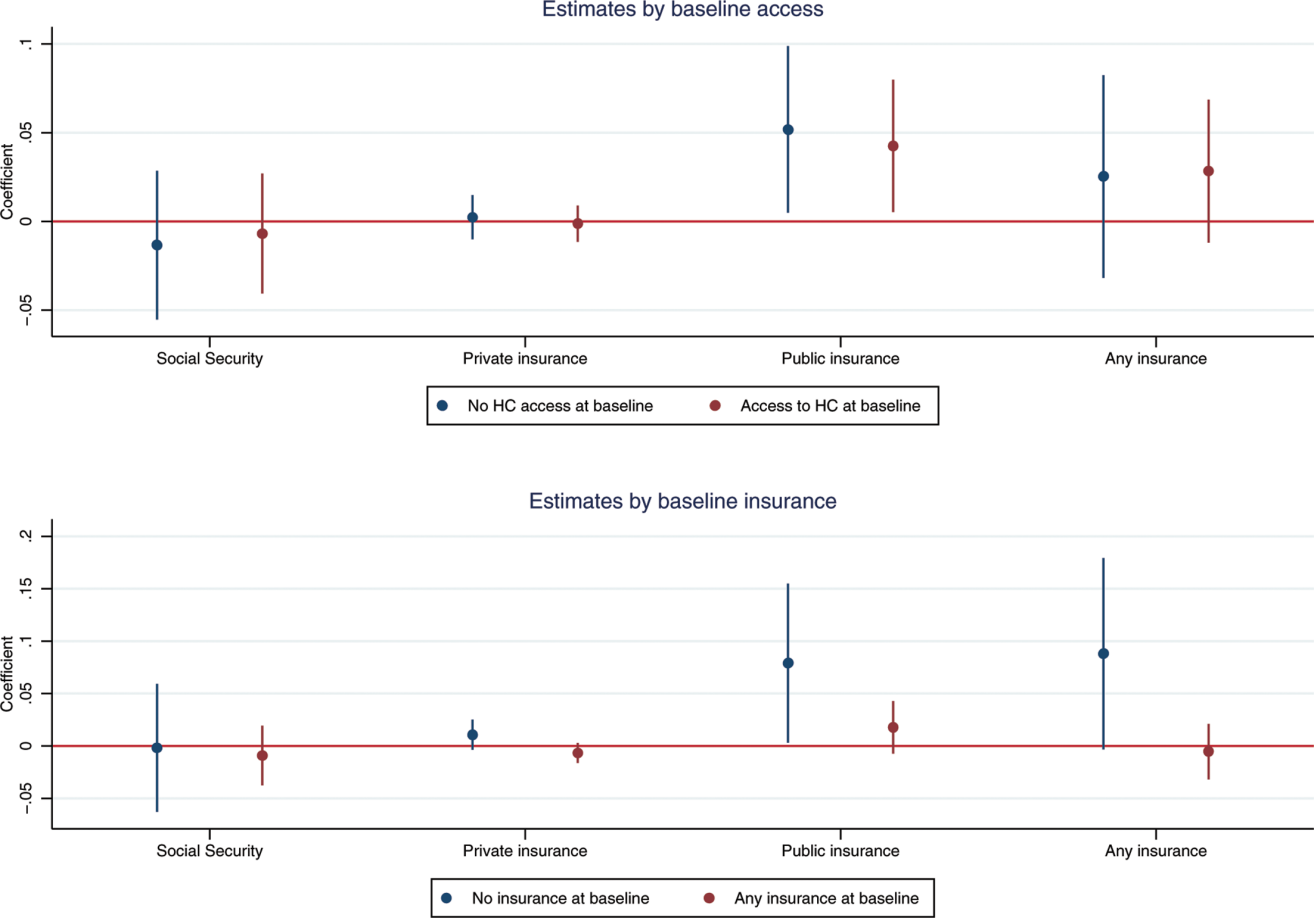
Impact of old-age pensions on health insurance uptake by baseline access and insurance coverage. HC, healthcare.

Figure 2 confirms that the old-age pension programme is not associated with changes in OOP expenditures, regardless of baseline status. As before, these results are explained by the impact of the pensions on health insurance uptake. The upper panel of figure 3 shows that the pension programme increased the uptake of public health insurance by 4.3 percentage points (95% CI 0.5 to 8.0) for individuals with healthcare utilisation at baseline and 5.2 percentage points (95% CI 0.5 to 9.9) for individuals without it. The lower panel shows that the pension scheme is associated with an 8.9 percentage point (95% CI −0.3 to 17.9) increase in insurance for individuals without baseline coverage. This is mostly driven by a 1.1 percentage point (95% CI −0.4 to 2.5) increase in private insurance and a 7.9 percentage point (95% CI 0.3 to 15.5) increase in public health insurance. For individuals with coverage, the pension scheme did not increase the overall take-up of health insurance, but the results suggest a shift from social security (beta=−0.009, 95% CI −0.037 to 0.019) and private insurance (beta=−0.007, 95% CI −0.016 to 0.003) to public insurance (beta=0.018, 95% CI −0.007 to 0.043).

We conducted a series of sensitivity analyses to verify the robustness of our results. One concern is potential differences in the village samples. While a difference-in-difference approach does not require balance at baseline, we conducted a matched difference in difference to verify the robustness of our results to baseline characteristics. Overall, the results yielded similar results (online supplementary appendix table 9 and appendix figures 6-8). We find similar results when evaluating those who reported taking up the old-age pension (online supplementary appendix table 10 and appendix figures 9-11). Finally, we evaluated the robustness of our SE estimation strategy by using clustered SEs at the individual level and reached the same conclusions as above (online supplementary appendix table 11 and appendix figures 12-14).

## Discussion

The results from our study suggest that old-age pension programmes can increase healthcare demand by increasing healthcare utilisation and health insurance uptake. Our findings support previous evidence that old-age pensions are associated with increased healthcare use.^29 30^ Our results also provide new insights on how such behaviours may differ by previous healthcare use. More specifically, we found that older individuals without any baseline use of healthcare increased their demand for basic informal healthcare, while those with some baseline use shift towards more formal care. This is in line with evidence from developed countries where the reduction of financial barriers leads to shifts to more expensive and usually higher quality care.^31 32^

Interestingly, we find no impact on OOP medical expenditures despite increased healthcare use, although our SEs here are large. This finding is at odds with descriptive research linking higher income to higher medical expenditures at both the microlevel and macrolevel in different contexts.^33 34^ One explanation could be that improvements in health due to the cash transfer lead to lower overall expenditures in healthcare.^35 36^ This is in line with current evidence on *Reconocer*,^19^ but the short time frame of this evaluation makes it unlikely. Another possibility is that higher uptake of health insurance protects individuals financially from higher healthcare use. Our findings support this explanation. The public health insurance scheme in Mexico, *Seguro Popular*, requires the payment of annual fees that can reach US$1370 (in 2014 PPP) for those in the highest income decile, but among our sample, we estimate that on average individuals would pay an annual health insurance premium of US$25. Consequently, it is possible that individuals use the *Reconocer* pension to pay for health insurance on top of other expenses.

Another aspect to note on the interaction with other programmes is that the implementation of *Reconocer* led to decreased claims for *Oportunidades*. Individuals could only participate in one of the programmes, and *Reconocer* provided larger amounts of income. From a policy perspective, this highlights the interaction that can occur between social programmes. It also implies that our estimates are a combination of individuals who only receive a marginal increase in income and those who receive a larger increase in income. As a consequence, the estimates are lower in magnitude than what they should be if no programme had been in place.

Alternatively, access to health insurance may enhance the effect of income programmes. *Seguro Popular*, for example, could substitute for income as a means for low-income households to gain healthcare. Moreover, income programmes may allow older adults to use health insurance by lowering non-price barriers, such as enabling recipients to pay transportation costs to healthcare facilities, as well as by improving affordability of medicine. Receiving a cash transfer may complement having access to health insurance and further boost the use of healthcare services. Hence, access to health insurance could be a mediator between the cash transfer programme and healthcare utilisation. Several studies have found that *Seguro Popular* has significant effects on healthcare use and ability to pay catastrophic expenditures,^37–41^ but there is limited or mixed evidence on its impacts for older adults. There is also evidence for the USA that access to health insurance increases healthcare utilisation for poorer individuals. The Oregon Experiment, for example, found that Medicaid coverage increased healthcare use, including use of preventive services and visits to emergency departments.^42 43^

There are several limitations to our work. First, even though the pension scheme was implemented experimentally, the use of only one control and one treatment location for our analysis poses some challenges to the appropriate estimation of SEs given the correlation of outcomes within villages. To address this, we followed current research recommendations and used bootstrapped SEs.^25^ We also presented in the online supplementary material results using clustered SEs at the individual level. Both estimation strategies yielded similar results and provide robustness to the conclusions drawn from our study.

Second, our study evaluates a short time period. Our results suggest that old-age pensions influence healthcare demand 6 months after implementation, but it would be of great policy interest to evaluate whether such effects persist.

Third, our self-reported measures of healthcare use, expenditures and access could suffer from measurement error. While the difference-in-difference approach would make this measurement error uncorrelated with the treatment status, it could reduce the precision of our estimates and explain the large SEs in some of our estimations.

Nevertheless, our overall results are in line with evidence from developed countries where income has been positively associated with insurance uptake.^44^ Furthermore, not all of the increase in healthcare utilisation that we find is attributable to health insurance uptake. Our findings suggest that individuals with baseline insurance coverage also increase their use of formal healthcare. The old-age pension may help such individuals to afford the copayments required for additional care^45 46^ or to cover non-medical costs necessary to access healthcare.^47 48^

Our study adds new insights to the literature concerning access to care. By exploiting the experimental implementation of an old-age pension programme with an individual fixed-effects difference-in-difference approach, we were able to reduce potential bias in the link between income and healthcare that can arise from self-selection and reverse causality. Our findings show a link between higher income and increased demand for healthcare, but they also suggest nuances in the relationship between healthcare and higher income in improving health. In particular, our results show that pensions may not just increase the amount of care but improve its quality as well. While pension income might be used for other purposes such as increasing food availability,^19^ our study shows that healthcare investments are still a priority for poor old age adults. In other settings where basic needs are already fulfilled, pensions may lead to even greater health investments.

Additionally, and more importantly, our results provide evidence on the complementarities that may occur between public pension and health programmes. It highlights that changes in one social programme could increase demand and use of another. The interaction between social programmes should be further studied as it may lead to a better design of social policies.

Finally, the emphasis of this study on the ageing population fills a gap in the literature. Our results show that, in contrast to the small effects theoretical literature would predict,^49^ cash transfers can increase use of healthcare even for older individuals.
