# Correction: The effect of old-age pensions on health care utilization patterns and insurance uptake in Mexico

**DOI:** 10.1136/bmjgh-2019-001771corr1

**Published:** 2019-12-11

Riumallo-Herl C, Aguila E. The effect of old-age pensions on health care utilization patterns and insurance uptake in Mexico. *BMJ Global Health* 2019;4:e001771. doi: 10.1136/bmjgh-2019-001771.

The license type for this paper has changed from CC BY-NC to CC BY.

