# Supplementary material for: The effect of old-age pensions on health care utilization patterns and insurance uptake in Mexico
Source: BMJ Glob Health. 2019 Nov 12;4(6):e001771. doi: 10.1136/bmjgh-2019-001771 (PMC6861075; doi:10.1136/bmjgh-2019-001771)
Supplement: Supplementary data [file bmjgh-2019-001771supp001.pdf]

Old-age pensions and health care

Appendix Figures

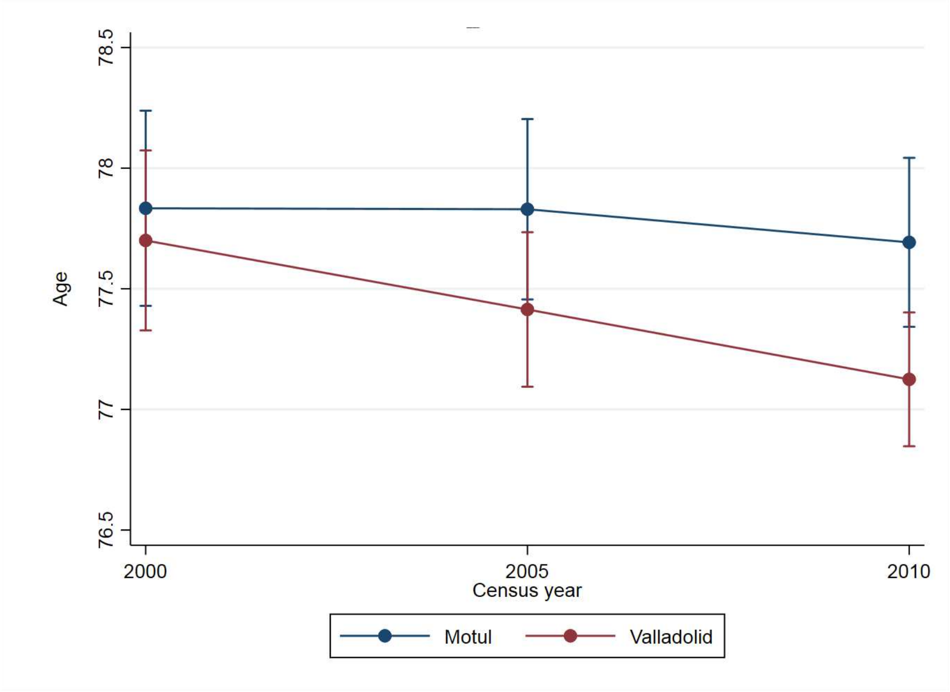

Appendix Figure 1. Descriptive statistics of age trends in Motul (control) and Valladolid (treatment)

Old-age pensions and health care

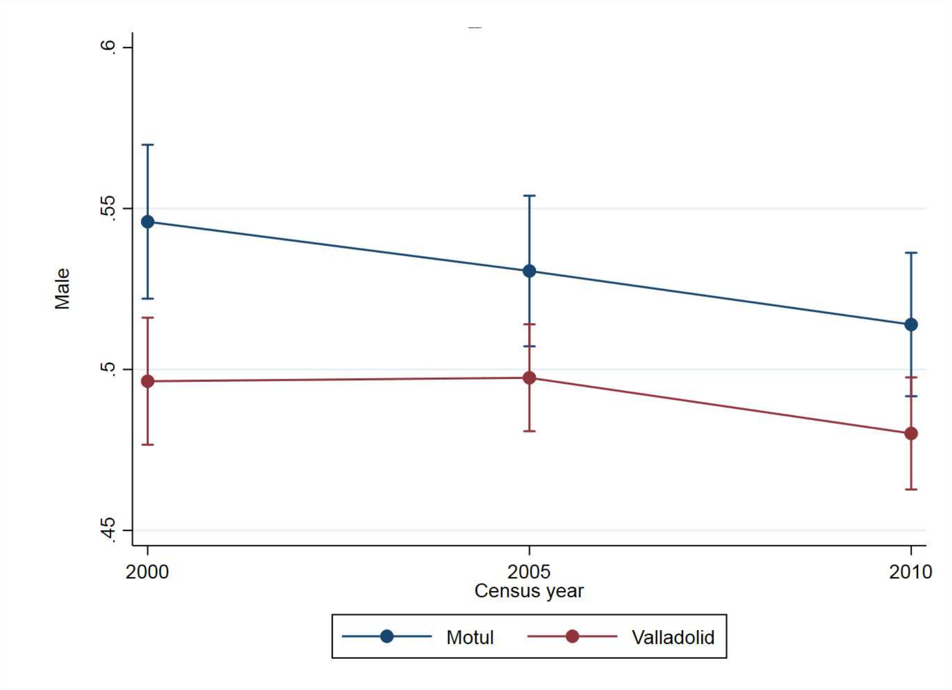

Appendix Figure 2. Descriptive statistics of gender trends in Motul (control) and Valladolid (treatment)

Old-age pensions and health care

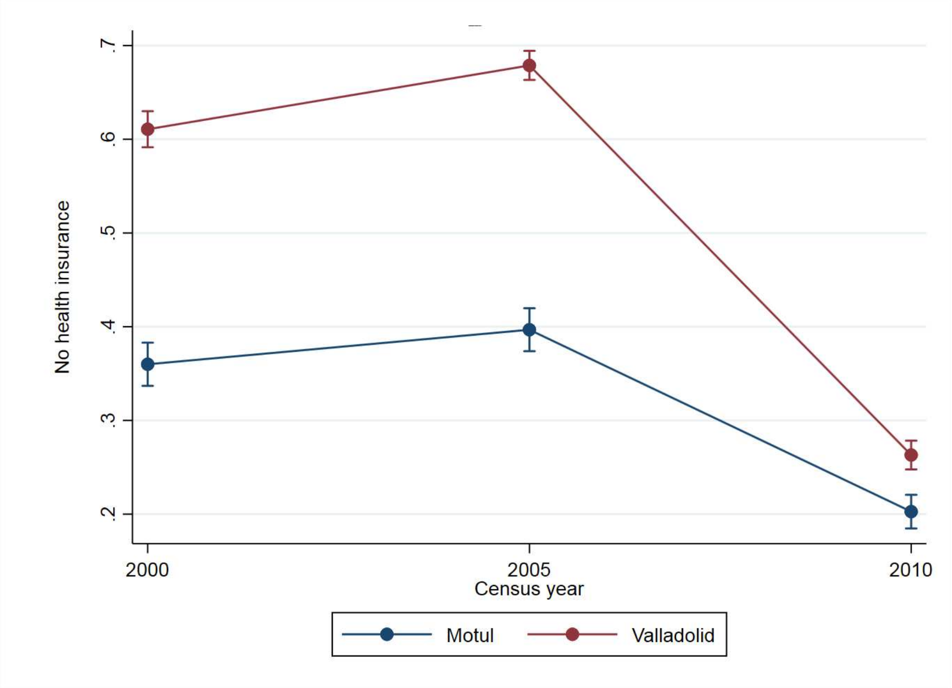

Appendix Figure 3. Descriptive statistics of health insurance coverage trends in Motul (control) and Valladolid (treatment)

Old-age pensions and health care

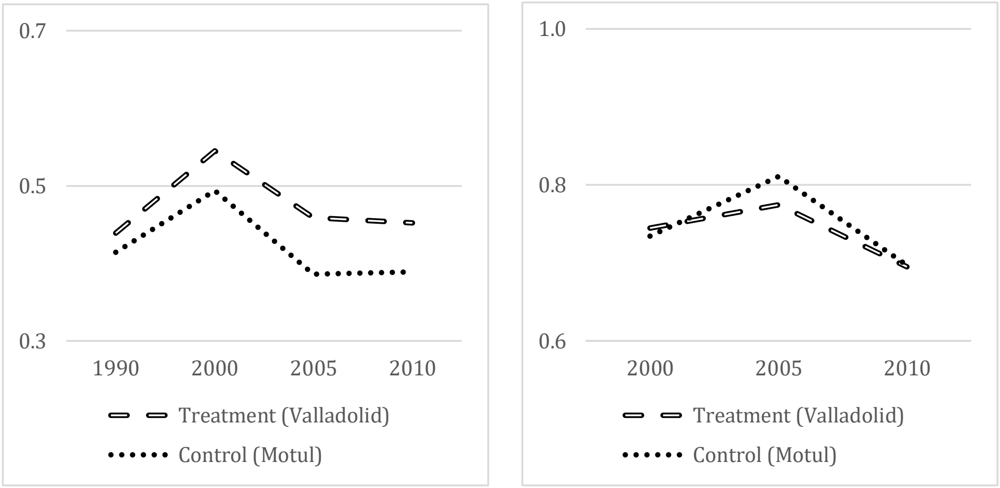

Gini Coefficient Human Development Index

Appendix Figure 4 Inequality and Human Development Indexes for Treatment and Control Groups

Notes: The Human Development Index (HDI) is a composite statistic of life expectancy, education, and income per capita. The minimum and maximum values of the indicator are set from 0 to 1 where 1 represents the highest observed values for the components of the indicator and 0 represents subsistence values. The Gini coefficient is a measure of statistical dispersion that represents the income distribution of a particular population. The range of values for this coefficient is from 0 to 1. Values closer to 0 represent more equality in the distribution of income and values closer to 1 represent higher inequality in the distribution of income.

Sources: United Nations Development Program for Human Development Index and Consejo Nacional de Evaluacion de la Politica de Desarrollo Social [CONEVAL] for Gini Coefficient.

Old-age pensions and health care

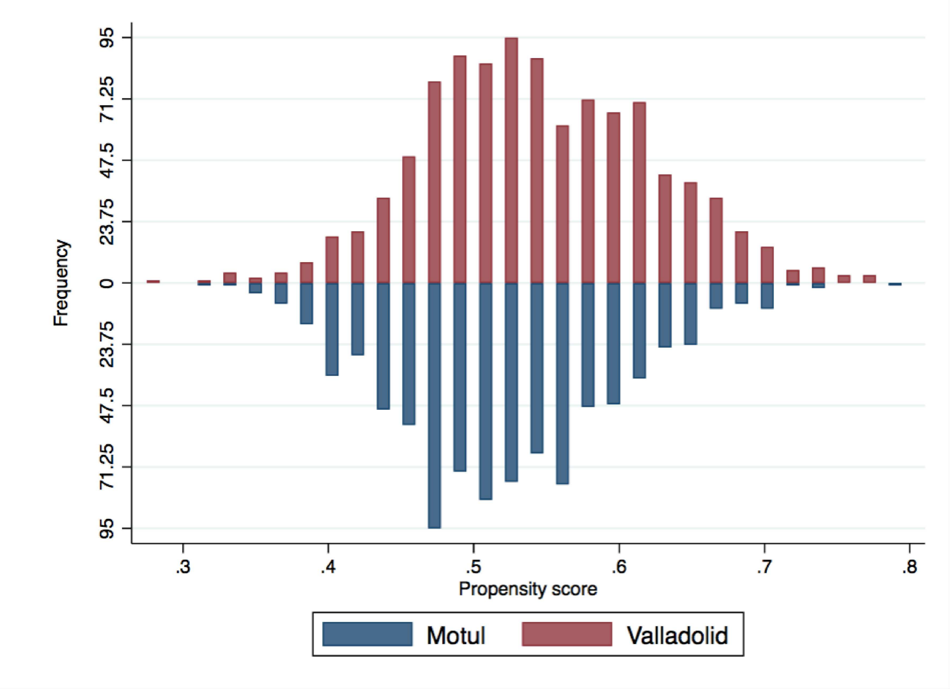

Appendix Figure 5. Propensity score estimates for Motul (control) and Valladolid (treatment)

Old-age pensions and health care

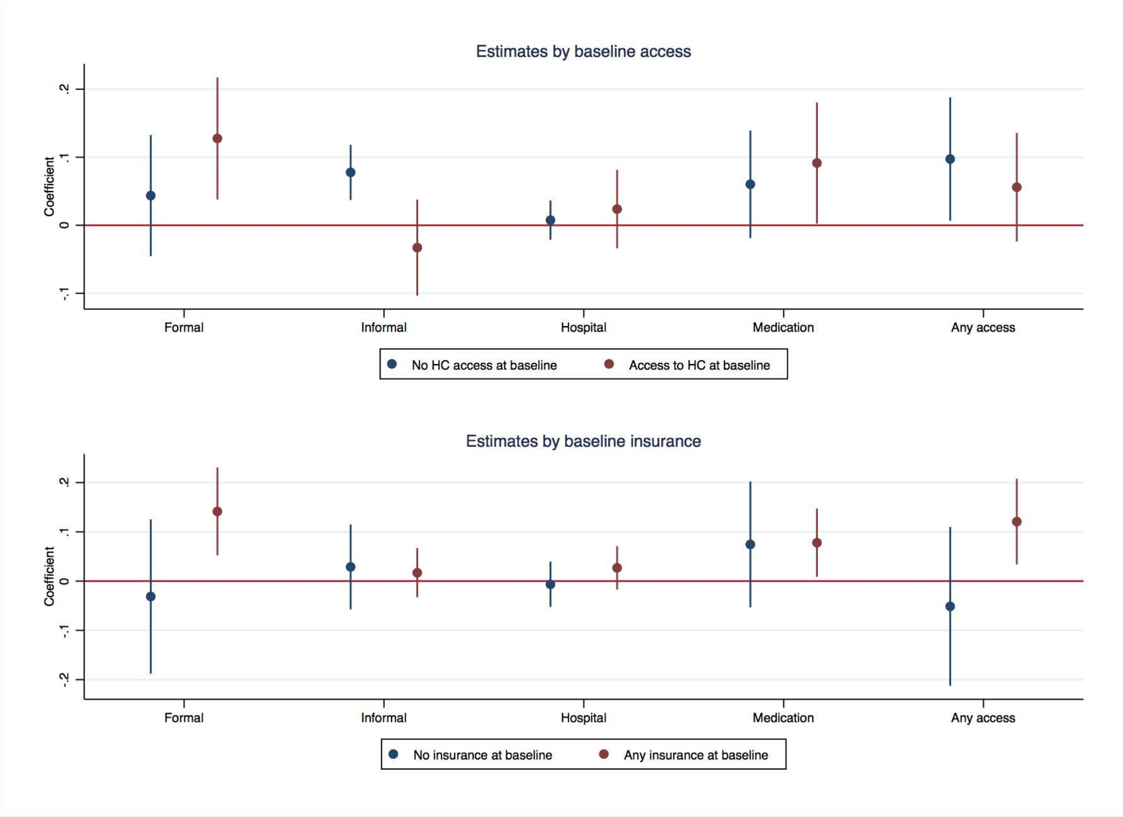

Appendix Figure 6 Impact of old-age pensions on health care utilization by baseline access and insurance coverage from matched difference-in-difference models

Old-age pensions and health care

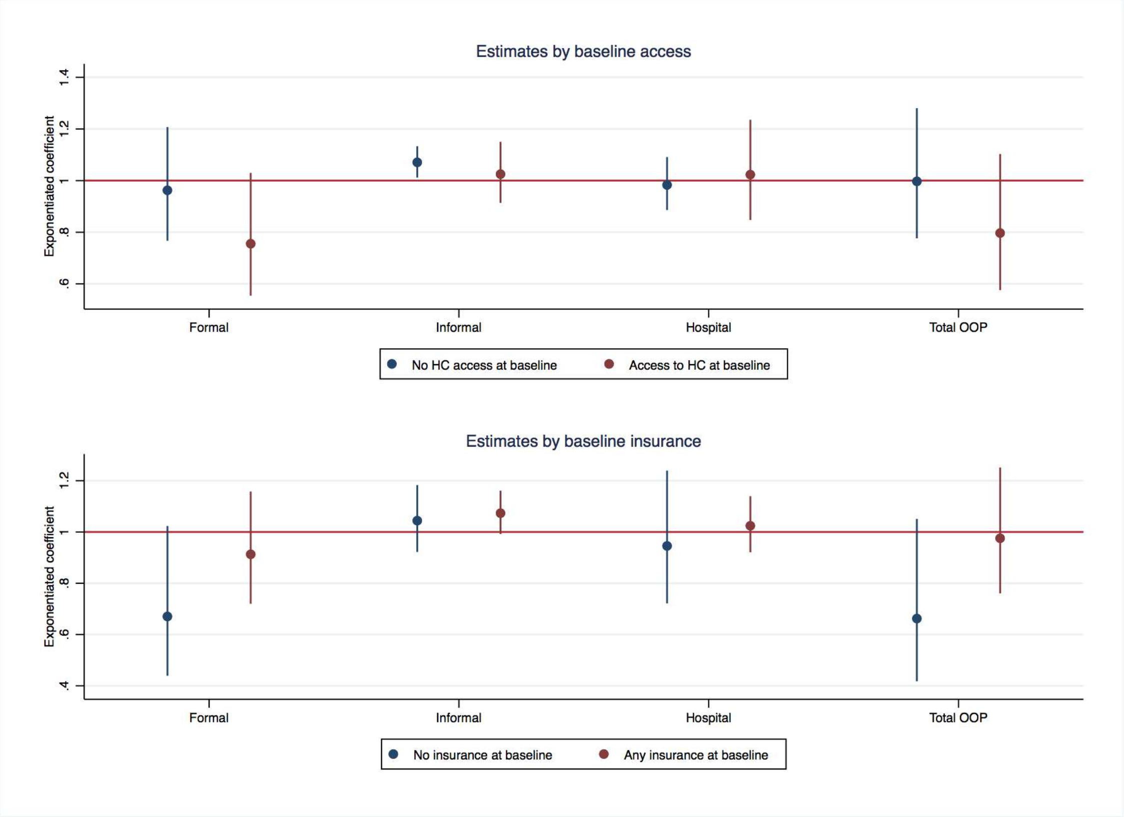

Appendix Figure 7 Impact of old-age pensions on health care expenditures by baseline access and insurance coverage from matched difference-in-difference models

Old-age pensions and health care

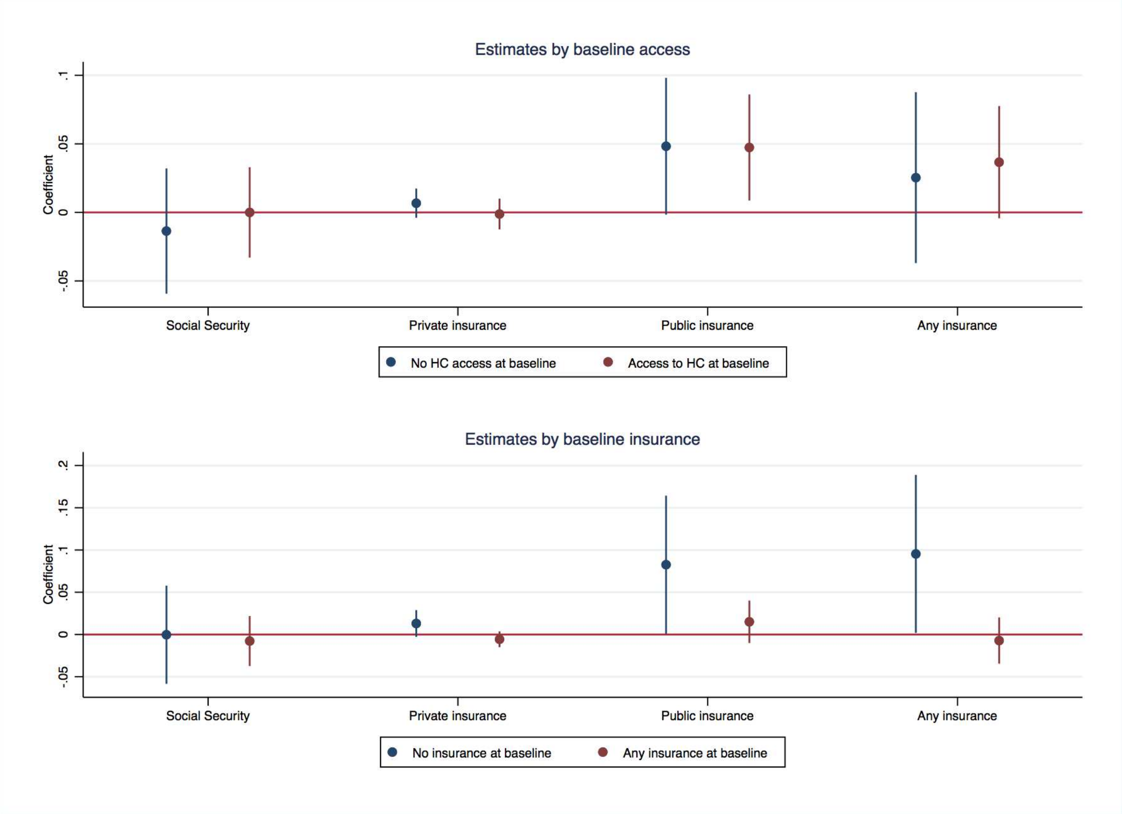

Appendix Figure 8 Impact of old-age pensions on health care insurance uptake by baseline access and insurance coverage from matched difference-in-difference models

Old-age pensions and health care

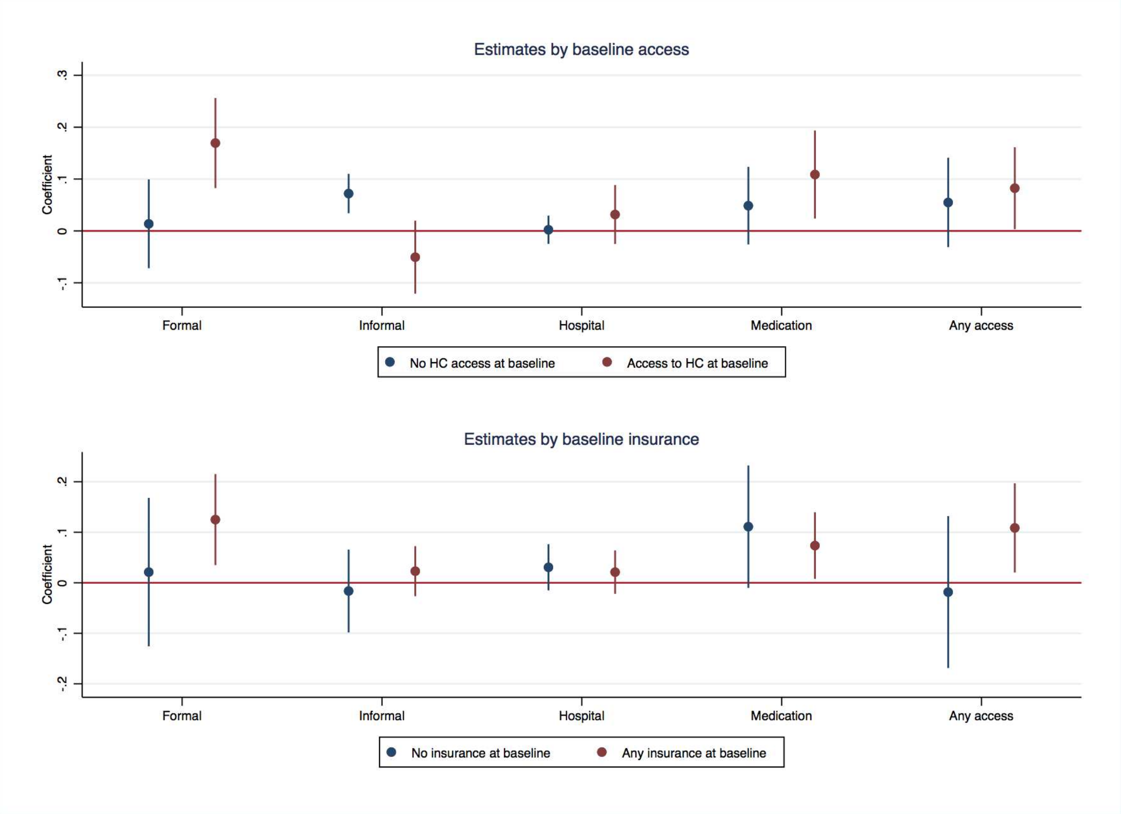

Appendix Figure 9 Impact of pension uptake on health care utilization by baseline access and insurance coverage

Old-age pensions and health care

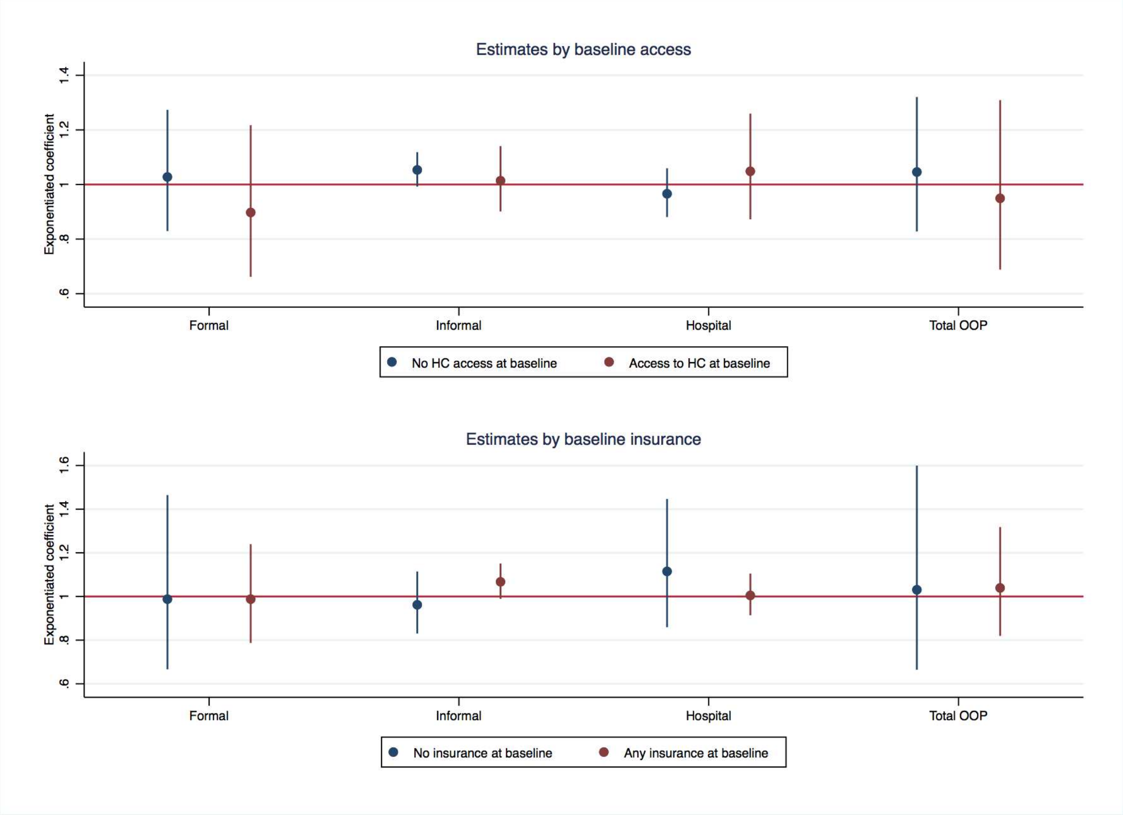

Appendix Figure 10 Impact of pension uptake on health care expenditure by baseline access and insurance coverage

Old-age pensions and health care

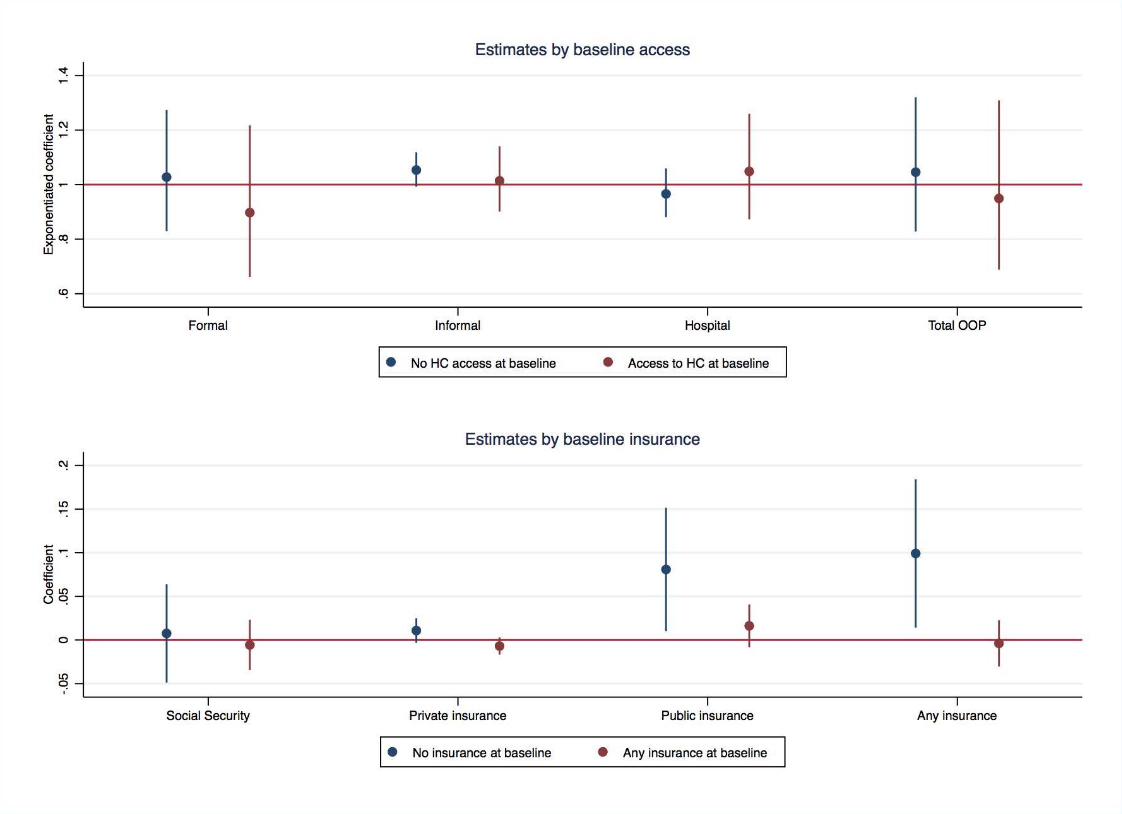

Appendix Figure 11 Impact of pension uptake on health insurance uptake by baseline access and insurance coverage

Old-age pensions and health care

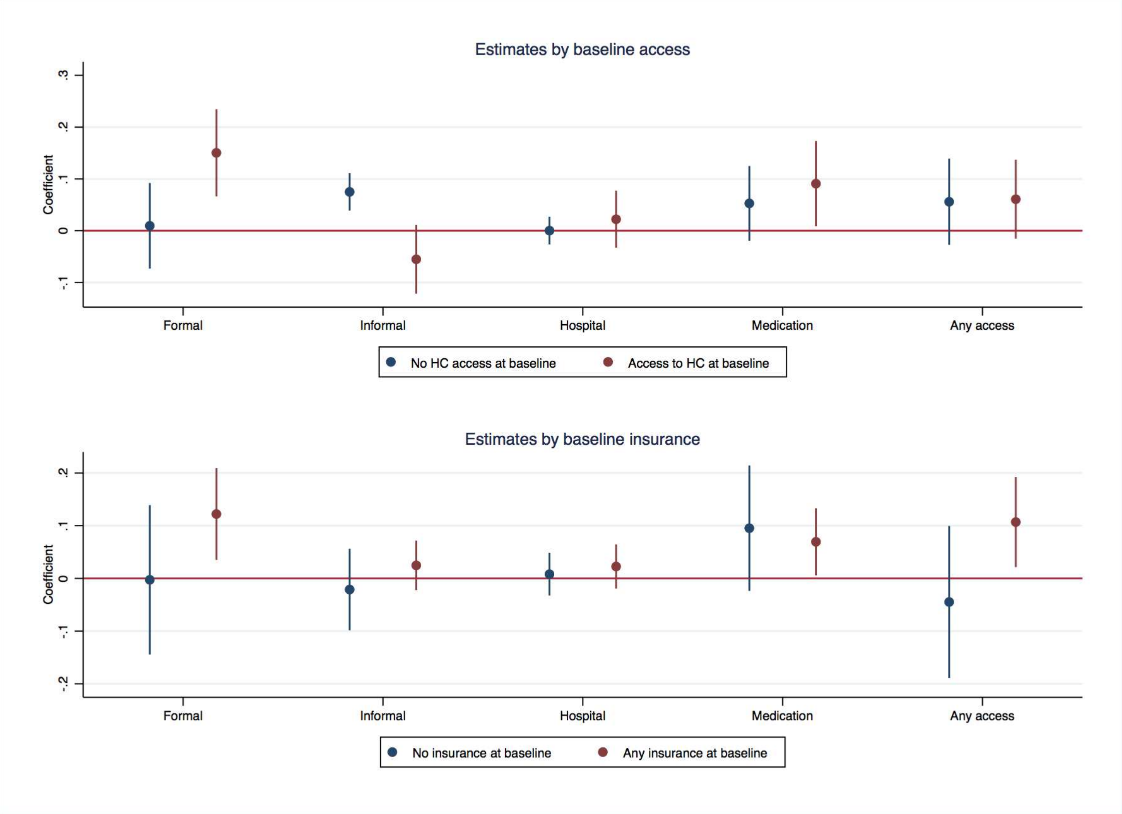

Appendix Figure 12 Impact of pension uptake on health care utilization by baseline access and insurance coverage using clustered standard errors at individual level

Old-age pensions and health care

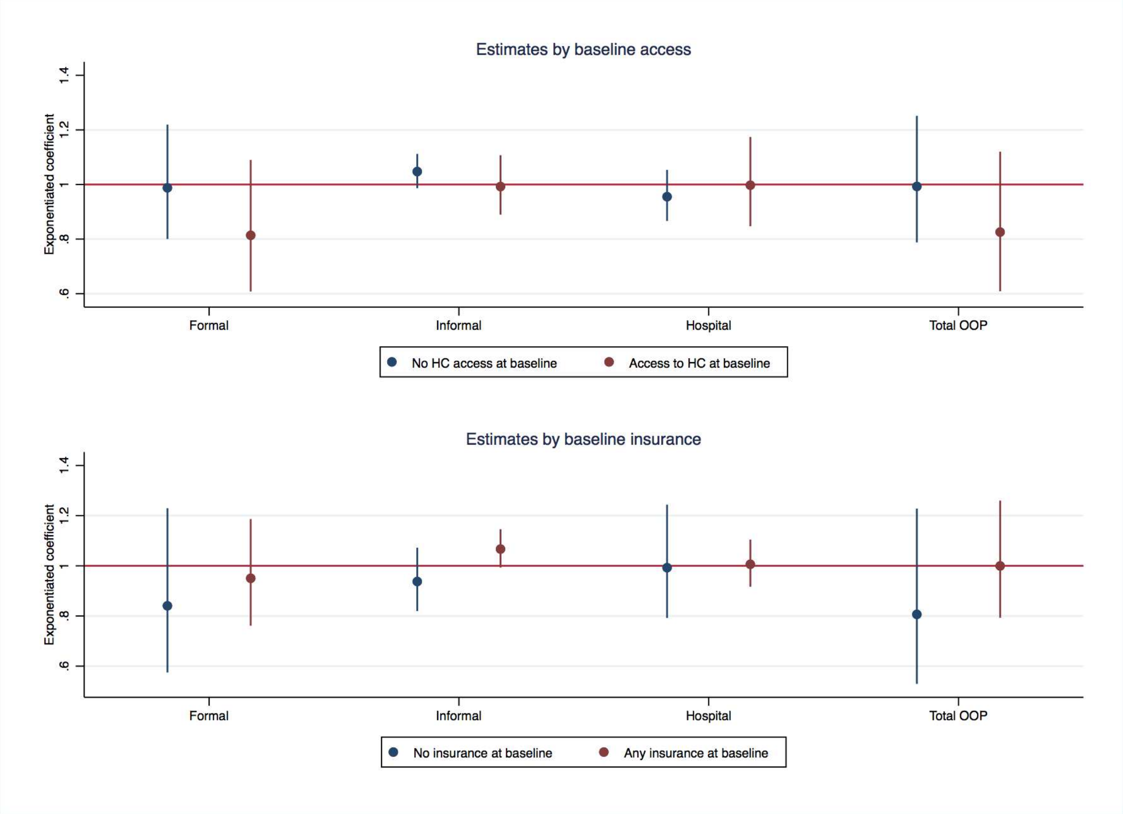

Appendix Figure 13 Impact of pension uptake on health care expenditure by baseline access and insurance coverage using clustered standard errors at the individual level

Old-age pensions and health care

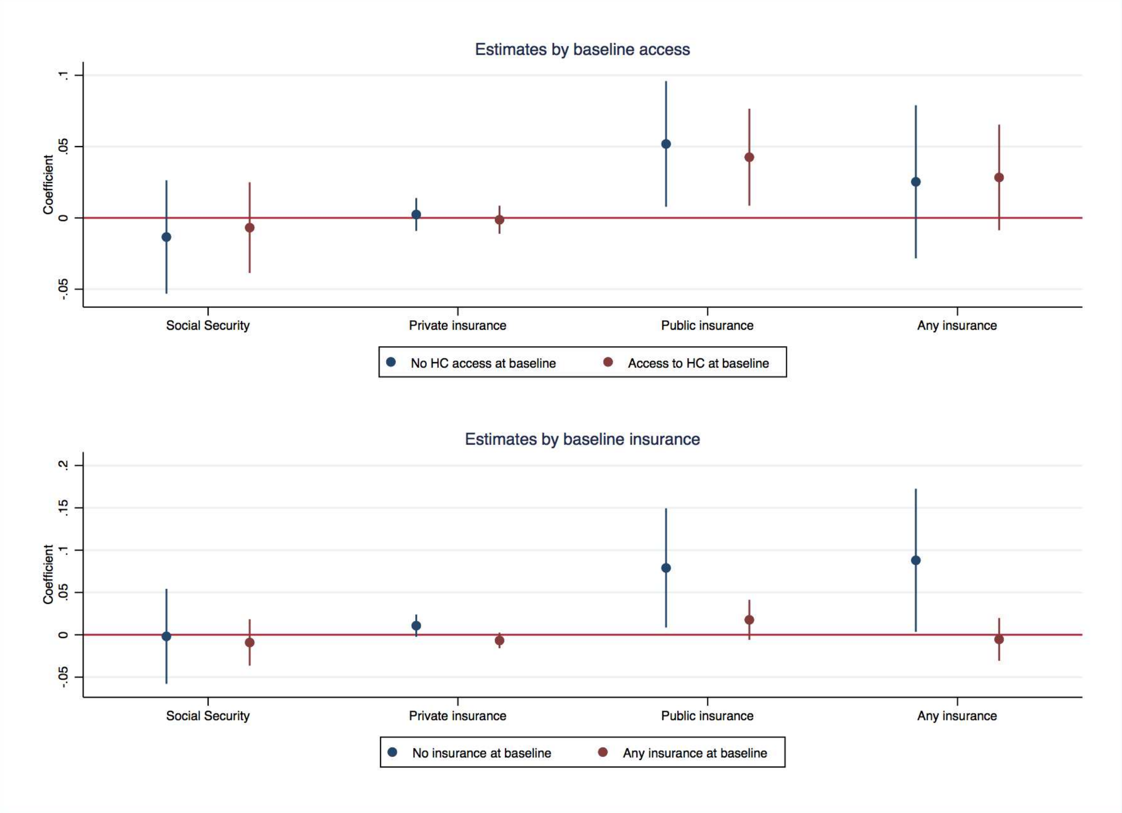

Appendix Figure 14 Impact of pension uptake on health insurance uptake by baseline access and insurance coverage using clustered standard errors at the individual level

Old-age pensions and health care

Appendix Tables

Appendix Table 1 Comparison of Valladolid and Motul with other localities with 20000 to 50000 inhabitants

	Valladolid (%)	Motul (%)	%P5	%P25	%P50	%P75	%P95
Households without earthen floor	93,9	96,0	79,0	88,6	92,4	95,0	97,1
Households with earthen floor	3,5	2,9	0,8	2,5	4,9	8,6	18,8
Households with access to potable water	91,7	88,9	60,5	87,4	93,7	95,9	97,3
Households with electricity	95,9	96,9	91,8	95,6	96,6	97,4	98,4
Households with television	90,8	91,9	86,4	91,1	94,1	95,3	96,9
Households with fridge	73,5	71,4	59,0	75,4	82,4	88,3	93,1
Households with washing machine	57,6	60,9	35,0	53,1	64,7	74,6	83,7

Old-age pensions and health care

Appendix Table 2 Comparison of ENCAHEY Screener in 2008 and Mexican Census 2005 and 2010

	Valladolid			Motul		
Sample	ENCAHEY 2008	Census 2005	Census 2010	ENCAHEY 2008	Census 2005	Census 2010
Total number of households	9,990	10,390	12,003	5,922	4,973	5,964
No. of households not contacted	342	--	--	266	--	--
No. of households refusing screener	1,644	--	--	1,594	--	--
No. of households without age-eligible persons	6,678	--	--	3,082	--	--
No. of age-eligible households	1,326	1,320	1,586	980	967	1,306
No. of age-eligible persons	1,594	1,601	1,628	1,131	1,266	1,922

Old-age pensions and health care

Appendix Table 3. Descriptive statistics of panel and baseline sample

	Treatment				Control					
	Baseline	Panel	Difference	P-value	Baseline	Panel	Difference	p-value	Diff-in-diff	P-value
Male	0.48	0.47	-0.011	0.786	0.49	0.52	0.032	0.440	0.043	0.228
SD	0.04	0.01	0.040		0.04	0.02	0.041		0.057	
Age	79.53	77.58	-1.960	0.001	78.70	77.60	-1.104	0.044	0.856	0.147
SD	0.57	0.19	0.604		0.50	0.22	0.546		0.814	
Education	1.96	1.76	-0.197	0.230	2.17	1.85	-0.315	0.040	-0.118	0.299
SD	0.15	0.06	0.164		0.14	0.06	0.152		0.224	
Couple	0.48	0.53	0.050	0.208	0.56	0.51	-0.047	0.250	-0.097	0.044
SD	0.04	0.01	0.040		0.04	0.02	0.041		0.057	
Divorced/Separated	0.04	0.03	-0.004	0.815	0.03	0.03	-0.001	0.945	0.003	0.449
SD	0.01	0.01	0.015		0.01	0.01	0.013		0.020	
Widow	0.41	0.38	-0.029	0.461	0.38	0.39	0.015	0.702	0.044	0.215
SD	0.04	0.01	0.039		0.04	0.02	0.040		0.056	
Household size	3.44	3.42	-0.029	0.869	3.30	3.48	0.181	0.272	0.210	0.190
SD	0.16	0.06	0.174		0.15	0.07	0.164		0.239	
Work for pay	0.17	0.17	-0.006	0.842	0.10	0.16	0.059	0.026	0.065	0.052
SD	0.03	0.01	0.030		0.02	0.01	0.026		0.040	
Household income	90.99	81.77	-9.220	0.446	150	922	82.94	76.61	-6.326	90.99
SD	11.66	3.09	12.066		3.75	3.13	4.886		13.018	
Chronic	0.83	0.75	-0.086	0.268	1.07	0.85	-0.214	0.008	-0.128	0.126
SD	0.07	0.03	0.077		0.07	0.03	0.081		0.112	
ADL Limitations	4.93	4.63	-0.298	0.381	4.83	4.80	-0.034	0.893	0.264	0.265
	0.32	0.12	0.339		0.18	0.18	0.250		0.421	
Reconocer	0.00	0.00	0.000		0.00	0.00	0.000		0.000	
SD	0.00	0.00	0.000		0.00	0.00	0.000		0.000	
Formal care utilization	0.45	0.46	0.010	0.805	0.55	0.52	-0.039	0.341	-0.049	0.195
SD	0.04	0.01	0.039		0.04	0.02	0.041		0.057	
Informal care utilization	0.12	0.12	-0.002	0.952	0.06	0.07	0.010	0.604	0.012	0.358
SD	0.02	0.01	0.026		0.02	0.01	0.020		0.033	
Hospital care utilization	0.12	0.05	-0.065	0.009	0.08	0.06	-0.023	0.301	0.042	0.104
SD	0.02	0.01	0.025		0.02	0.01	0.022		0.033	
Medication adherence	0.79	0.76	-0.035	0.276	0.83	0.82	-0.012	0.696	0.023	0.303
SD	0.03	0.01	0.032		0.03	0.01	0.031		0.045	
Formal expenditures	13.13	11.72	-1.417	0.703	21.42	8.27	-13.153	0.198	-11.736	0.139
SD	3.50	1.23	3.707		10.00	1.93	10.181		10.835	
Informal expenditures	1.45	1.02	-0.432	0.662	0.55	0.15	-0.393	0.409	0.039	0.486
SD	0.94	0.32	0.988		0.47	0.05	0.474		1.096	
Hospital expenditures	41.07	24.03	-17.046	0.616	1.47	4.36	2.899	0.269	19.944	0.279
SD	32.19	10.90	33.984		1.01	2.42	2.622		34.085	
Total OOP expenditures	55.66	36.76	-18.895	0.583	23.43	12.79	-10.647	0.319	8.248	0.409
SD	32.47	11.32	34.387		10.05	3.55	10.657		36.000	
Social security	0.42	0.39	-0.022	0.582	0.63	0.64	0.012	0.759	0.034	0.272
SD	0.04	0.01	0.039		0.04	0.02	0.040		0.056	
Private insurance	0.04	0.03	-0.009	0.551	0.02	0.00	-0.014	0.149	-0.005	0.387
SD	0.01	0.01	0.015		0.01	0.00	0.010		0.018	
Public insurance	0.21	0.28	0.075	0.025	0.06	0.11	0.049	0.016	-0.025	0.257
SD	0.03	0.01	0.033		0.02	0.01	0.020		0.039	
Health insurance	0.66	0.68	0.027	0.471	0.70	0.74	0.038	0.316	0.010	0.422
SD	0.03	0.01	0.037		0.03	0.02	0.037		0.053	
Observations	187	1120			182	791				

Old-age pensions and health care

Appendix Table 4. Descriptive statistics of panel sample and those that die between first and second wave

	Treatment				Control					
	Dead	Panel	Difference	P-value	Dead	Panel	Difference	P-value	Diff-in-diff	P-value
Male	0.49	0.47	-0.027	0.674	0.44	0.52	0.082	0.235	0.109	0.122
SD	0.06	0.01	0.064		0.07	0.02	0.069		0.094	
Age	84.35	77.58	-6.779	0.000	81.46	77.60	-3.857	0.000	2.922	0.019
SD	1.00	0.19	1.019		0.95	0.22	0.979		1.413	
Education	1.83	1.76	-0.065	0.811	1.71	1.85	0.137	0.576	0.203	0.290
SD	0.26	0.06	0.271		0.24	0.06	0.245		0.365	
Couple	0.45	0.53	0.085	0.187	0.47	0.51	0.040	0.568	-0.046	0.314
SD	0.06	0.01	0.064		0.07	0.02	0.069		0.094	
Divorced/Separated	0.02	0.03	0.019	0.259	0.04	0.03	-0.009	0.736	-0.027	0.184
SD	0.02	0.01	0.016		0.02	0.01	0.025		0.030	
Widow	0.49	0.38	-0.109	0.093	0.46	0.39	-0.062	0.373	0.048	0.307
SD	0.06	0.01	0.064		0.07	0.02	0.069		0.094	
Household size	3.62	3.42	-0.200	0.428	3.82	3.48	-0.347	0.224	-0.146	0.349
SD	0.24	0.06	0.251		0.27	0.07	0.282		0.378	
Work for pay	0.08	0.17	0.088	0.014	0.09	0.16	0.075	0.064	-0.013	0.404
SD	0.03	0.01	0.035		0.04	0.01	0.040		0.053	
Household income	87.12	81.77	-5.351	0.809	78.72	76.61	-2.113	0.663	3.238	0.443
	21.78	3.09	22.000		3.69	3.13	4.842		22.527	
Chronic	0.92	0.75	-0.175	0.187	1.23	0.85	-0.376	0.012	-0.201	0.152
SD	0.13	0.03	0.131		0.14	0.03	0.146		0.196	
ADL Score	6.73	4.63	-2.099	0.002	5.06	4.80	-0.255	0.345	1.844	0.004
	0.62	0.12	0.629		0.21	0.18	0.270		0.685	
Reconocer	0.00	0.00	0.000		0.00	0.00	0.000		0.000	
SD	0.00	0.00	0.000		0.00	0.00	0.000		0.000	
Formal care utilization	0.46	0.46	0.003	0.966	0.56	0.52	-0.046	0.509	-0.048	0.303
SD	0.06	0.01	0.064		0.07	0.02	0.069		0.094	
Informal care utilization	0.12	0.12	-0.002	0.969	0.05	0.07	0.018	0.562	0.020	0.353
SD	0.04	0.01	0.042		0.03	0.01	0.031		0.052	
Hospital care utilization	0.22	0.05	-0.165	0.003	0.14	0.06	-0.081	0.092	0.084	0.116
SD	0.05	0.01	0.053		0.05	0.01	0.047		0.071	
Medication adherence	0.80	0.76	-0.044	0.397	0.79	0.82	0.028	0.620	0.072	0.173
SD	0.05	0.01	0.052		0.05	0.01	0.056		0.076	
Formal expenditures	17.96	11.72	-6.246	0.480	7.09	8.27	1.184	0.676	7.430	0.211
SD	8.71	1.23	8.801		2.07	1.93	2.829		9.245	
Informal expenditures	3.58	1.02	-2.559	0.344	1.59	0.15	-1.439	0.343	1.121	0.358
SD	2.67	0.32	2.686		1.50	0.05	1.503		3.078	
Hospital expenditures	17.72	24.03	6.307	0.641	1.08	4.36	3.287	0.215	-3.020	0.413
SD	8.00	10.90	13.518		1.08	2.42	2.647		13.775	
Total OOP expenditures	39.26	36.76	-2.498	0.882	9.75	12.79	3.033	0.504	5.531	0.375
SD	12.37	11.32	16.768		2.82	3.55	4.536		17.370	
Social security	0.35	0.39	0.040	0.520	0.71	0.64	-0.066	0.311	-0.105	0.118
SD	0.06	0.01	0.062		0.06	0.02	0.064		0.089	
Private insurance	0.03	0.03	-0.002	0.945	0.02	0.00	-0.016	0.396	-0.014	0.312
SD	0.02	0.01	0.022		0.02	0.00	0.018		0.029	
Public insurance	0.32	0.28	-0.041	0.498	0.04	0.11	0.069	0.015	0.110	0.049
SD	0.06	0.01	0.060		0.03	0.01	0.028		0.066	
Health insurance	0.69	0.68	-0.007	0.900	0.75	0.74	-0.014	0.821	-0.006	0.471
SD	0.06	0.01	0.059		0.06	0.02	0.060		0.084	
Observations	65	1120			57	791				

Old-age pensions and health care

Appendix Table 5 OLS Regression of common trends assumption for OOP expenditures using data from ENIGH survey in Mexico

	OOP health expenditure per capita	Primary care and hospitalization expenditure per capita
Valladolid	80.8251	105.2127
	(181.1550)	(161.3020)
Interaction terms		
Valladolid *2005	348.9858	322.1923
	(331.2560)	(309.7650)
Valladolid *2006	116.1298	91.4556
	(212.9740)	(195.6610)
Valladolid *2008	313.6093	256.9465
	(240.4440)	(217.3310)
Valladolid *2010	64.8180	29.7004
	(230.8130)	(214.0690)
Year		
2005	-91.0360	-54.8583
	(141.4500)	(123.8450)
2006	-146.8766	-96.2453
	(135.8780)	(119.8490)
2008	-206.5759	-160.3200
	(131.1150)	(112.3300)
2010	-41.9065	10.6572
	(149.1750)	(134.3540)
Constant	277.2063**	207.1878*
	(127.3600)	(109.4430)
F (interaction)	0.6731	0.6200
Prob > F (interaction)	0.6112	0.6486

Note: Robust standard errors in parenthesis. The model before compares the trends of health care expenditure in treatment and control villages using data from the Mexican Household Economic Survey from 2004 until 2010. The year 2004 was used as the reference category.

[*] $p < .1$, [**] $p < .05$, [***] $p < .01$

Old-age pensions and health care

Appendix Table 6 OLS Regression of common trends assumption for poverty variables using data from Census

	Earthen floor	Number of rooms	Access to potable water	Sewage	Electricity	Lives alone
Valladolid	0.0753***	0.1490**	-0.0043	-0.0033	-0.0076	-0.0412**
	(0.0251)	(0.0716)	(0.0044)	(0.0086)	(0.0152)	(0.0168)
Interaction terms						
Valladolid * 1995	0.1260	-0.5860	0.0057	0.0030	0.0045	0.0054
	(0.1590)	(0.6370)	(0.0053)	(0.0088)	(0.0184)	(0.0239)
Valladolid * 2000	-0.0273	0.0327	0.0051	0.0018	0.0118	0.0135
	(0.0413)	(0.1700)	(0.0060)	(0.0095)	(0.0174)	(0.0237)
Valladolid * 2005	0.0498	0.0097	0.0058	0.0049	0.0137	0.0129
	(0.0385)	(0.1780)	(0.0046)	(0.0086)	(0.0169)	(0.0228)
Valladolid * 2010	0.0678	-0.0941	0.0067	0.0042	0.0057	-0.00386
	(0.0676)	(0.4290)	(0.0054)	(0.0087)	(0.0162)	(0.0227)
Year						
1995	-0.1340	0.5880	-0.0055	-0.0243***	0.0376***	0.0226
	(0.1070)	(0.5700)	(0.0043)	(0.0067)	(0.0135)	(0.0190)
2000	-0.1800***	0.3333***	-0.0019	-0.0179**	0.0472***	0.0255
	(0.0277)	(0.1160)	(0.0047)	(0.0073)	(0.0128)	(0.0186)
2005	-0.2090***	0.4250***	-0.0084**	-0.0257***	0.0483***	0.0317*
	(0.0250)	(0.1270)	(0.0037)	(0.0066)	(0.0126)	(0.0182)
2010	-0.2120***	1.4150***	-0.0037	-0.0247***	0.0617***	0.0648***
	(0.0380)	(0.2860)	(0.0044)	(0.0066)	(0.0119)	(0.0183)
Constant	0.2460***	2.4120***	0.0084**	0.0257***	0.9200***	0.1230***
	(0.0191)	(0.0504)	(0.0037)	(0.0066)	(0.0111)	(0.0134)
F (interaction)	0.1520	0.5439	0.5439	0.4792	0.3349	0.1520
Prob > F (interaction)	0.9284	0.6522	0.6522	0.6967	0.8001	0.9284

Note: Robust standard errors in parentheses. The year 1990 was the reference category for lives alone, earthen floor, total number of rooms, access to potable water, sewage septic tank, sewage to lake, sewage not available, and electricity. Source: Census 1990, 1995, 2005, and 2010.

* $p < .1$, ** $p < .05$, *** $p < .01$

Old-age pensions and health care

Appendix Table 7 Characteristics of population in treatment and control villages from 2005 census

Variable (% or index)	Treatment (Valladolid)	Control (Motul)
Illiterate population 15 years old or above	10.9	11.2
Households without electricity	2.1	2.8
Households with earthen floor	3.6	2.9
Households without refrigerator	24.9	28.3
Poverty index	-1.1	-0.9

Old-age pensions and health care

Appendix Table 8 Odds ratio of the old-age pension program on health care utilization and insurance uptake

	(1)	(2)	(3)	(4)	(5)
Panel A: Health care utilization					
	Formal care	Informal care	Hospital stays	Medication	Any access
Treatment*Post	1.651	1.091	1.430	2.225	1.399
	[1.192,2.286]	[0.606,1.964]	[0.693,2.948]	[1.453,3.408]	[1.016,1.928]
Post	0.978	0.746	0.614	1.109	0.972
	[0.748,1.278]	[0.444,1.253]	[0.339,1.113]	[0.782,1.573]	[0.746,1.265]
Observations	3855	3855	3854	3852	3855
Individuals	2432	2432	2431	2431	2432
Panel B: Health insurance uptake					
	Social security	Private HI	Public HI	Any HI	
Treatment*Post	0.721		3.833	1.441	
	[0.261,1.992]		[1.280,11.478]	[0.615,3.376]	
Wave 2	1.100		2.000	1.818	
	[0.467,2.590]		[0.856,4.673]	[0.871,3.795]	
Observations	4060	4060	4060	4060	
Individuals	2487	2487	2487	2487	

Note: 95% Confidence intervals are obtained using bootstrapped standard errors. The coefficients are estimated with a matched individual fixed effect difference-in-difference models controlling for changes in age fixed effects, education years, marital status, household size, labour force status, chronic conditions, and number of limitations in daily living activities. Valladolid is the treatment village where the old-age pension scheme was implemented in December 2008. The models use data from the baseline and follow-up survey.

Old-age pensions and health care

Appendix Table 9 Effect of the old-age pension program on health care utilization, health expenditures, and insurance uptake using matched difference-in-differences

	(1)	(2)	(3)	(4)	(5)
Panel A: Health care utilization					
	Formal care	Informal care	Hospital stays	Medication	Any access
Treatment*Post	0.079	0.016	0.017	0.081	0.066
	[0.002,0.156]	[-0.027,0.059]	[-0.016,0.051]	[0.020,0.141]	[-0.011,0.143]
Post	0.070	0.108	-0.071	-0.171	0.063
	[-0.277,0.417]	[-0.109,0.326]	[-0.212,0.070]	[-0.383,0.041]	[-0.285,0.411]
Observations	3033	3033	3032	3031	3033
Individuals	1811	1811	1810	1810	1811
Panel B: Health care expenditures [Log+1]					
	Formal care	Informal care	Hospital stays	Total OOP	
Treatment*Post	-0.210	0.047	0.004	-0.162	
	[-0.414,-0.005]	[-0.018,0.112]	[-0.103,0.112]	[-0.381,0.057]	
Wave 2	0.966	0.323	-0.113	1.174	
	[0.225,1.708]	[-0.224,0.870]	[-0.375,0.149]	[0.283,2.065]	
Observations	3033	3033	3033	3033	
Individuals	1811	1811	1811	1811	
Panel C: Health insurance uptake					
	Social security	Private HI	Public HI	Any HI	
Treatment*Post	-0.003	0.001	0.041	0.032	
	[-0.030,0.024]	[-0.006,0.009]	[0.012,0.071]	[-0.003,0.068]	
Wave 2	-0.018	-0.010	-0.007	-0.040	
	[-0.094,0.057]	[-0.030,0.011]	[-0.076,0.063]	[-0.132,0.052]	
Observations	3026	3026	3026	3026	
Individuals	1811	1811	1811	1811	

Note: 95% Confidence intervals are obtained using bootstrapped standard errors. The coefficients are estimated with a matched individual fixed effect difference-in-difference models controlling for changes in age fixed effects, education years, marital status, household size, labour force status, chronic conditions, and number of limitations in daily living activities. Valladolid is the treatment village where the old-age pension scheme was implemented in December 2008. The models use data from the baseline and follow-up survey.

Old-age pensions and health care

Appendix Table 10 Effect of the pension uptake on health care utilization, health expenditures, and insurance uptake

	(1)	(2)	(3)	(4)	(5)
Panel A: Health care utilization					
	Formal care	Informal care	Hospital stays	Medication	Any access
Treatment*Post	0.090	0.009	0.014	0.077	0.068
	[0.015,0.164]	[-0.032,0.050]	[-0.019,0.047]	[0.020,0.134]	[-0.006,0.143]
Post	0.042	0.016	-0.078	-0.146	0.009
	[-0.261,0.345]	[-0.188,0.220]	[-0.220,0.065]	[-0.392,0.100]	[-0.300,0.319]
Observations	3818	3818	3817	3815	3818
Individuals	2397	2397	2396	2396	2397
Panel B: Health care expenditures [Log]					
	Formal care	Informal care	Hospital stays	Total OOP	
Treatment*Post	-0.120	0.024	-0.013	-0.100	
	[-0.311,0.071]	[-0.040,0.089]	[-0.109,0.082]	[-0.305,0.105]	
Wave 2	0.841	0.227	-0.145	0.975	
	[0.179,1.503]	[-0.248,0.702]	[-0.396,0.106]	[0.181,1.768]	
Observations	3818	3818	3818	3818	
Individuals	2397	2397	2397	2397	
Panel C: Health insurance uptake					
	Social security	Private HI	Public HI	Any HI	
Treatment*Post	-0.006	-0.000	0.042	0.031	
	[-0.032,0.020]	[-0.008,0.007]	[0.015,0.069]	[-0.002,0.063]	
Wave 2	-0.021	-0.008	-0.011	-0.048	
	[-0.089,0.046]	[-0.028,0.011]	[-0.077,0.055]	[-0.136,0.041]	
Observations	3751	3751	3751	3751	
Individuals	2367	2367	2367	2367	

Note: 95% Confidence intervals are obtained using bootstrapped standard errors. The coefficients are estimated with an individual fixed effect difference-in-difference models controlling for changes in age fixed effects, education years, marital status, household size, labour force status, chronic conditions, and number of limitations in daily living activities. Valladolid is the treatment village where the old-age pension program was implemented in December 2008. The models use data from the baseline and follow-up survey.

Old-age pensions and health care

Appendix Table 11 Effect of the pension uptake on health care utilization, health expenditures, and insurance uptake using standard errors clustered at individual level

	(1)	(2)	(3)	(4)	(5)
Panel A: Health care utilization					
	Formal care	Informal care	Hospital stays	Medication	Any access
Treatment*Post	0.090	0.009	0.014	0.077	0.068
	[0.017,0.162]	[-0.031,0.049]	[-0.018,0.046]	[0.021,0.132]	[-0.004,0.141]
Post	0.042	0.016	-0.078	-0.146	0.009
	[-0.242,0.326]	[-0.174,0.206]	[-0.210,0.054]	[-0.374,0.083]	[-0.280,0.299]
Observations	3818	3818	3817	3815	3818
Individuals	2397	2397	2396	2396	2397
Panel B: Health care expenditures [Log]					
	Formal care	Informal care	Hospital stays	Total OOP	
Treatment*Post	-0.120	0.024	-0.013	-0.100	
	[-0.306,0.066]	[-0.038,0.087]	[-0.106,0.080]	[-0.299,0.099]	
Wave 2	0.841	0.227	-0.145	0.975	
	[0.219,1.463]	[-0.214,0.668]	[-0.384,0.095]	[0.231,1.718]	
Observations	3818	3818	3818	3818	
Individuals	2397	2397	2397	2397	
Panel C: Health insurance uptake					
	Social security	Private HI	Public HI	Any HI	
Treatment*Post	-0.006	-0.000	0.042	0.031	
	[-0.031,0.019]	[-0.008,0.007]	[0.016,0.068]	[-0.001,0.062]	
Wave 2	-0.021	-0.008	-0.011	-0.048	
	[-0.086,0.043]	[-0.027,0.010]	[-0.075,0.053]	[-0.132,0.037]	
Observations	3751	3751	3751	3751	
Individuals	2367	2367	2367	2367	

Note: 95% Confidence intervals are obtained using standard errors clustered at individual level. The coefficients are estimated with an individual fixed effect difference-in-difference models controlling for changes in age fixed effects, education years, marital status, household size, labour force status, chronic conditions, and number of limitations in daily living activities. Valladolid is the treatment village where the old-age pension program was implemented in December 2008. The models use data from the baseline and follow-up survey.
